# T helper 17 cells and group 3 innate lymphoid cells define a spectrum of psoriasis endotypes

**DOI:** 10.3389/fimmu.2026.1880068

**Published:** 2026-07-02

**Authors:** Michael P. Schön

**Affiliations:** Department of Dermatology, Venereology and Allergology, University Medical Center Göttingen, Göttingen, Germany

**Keywords:** endotype, IL-17, IL-23, immune regulation, innate lymphoid cells (ILCs), psoriasis, Th17

## Abstract

Psoriasis is a prototypical chronic inflammatory skin disease that illustrates fundamental interactions between innate and adaptive immune pathways. Central to its pathophysiology is the interleukin-23/interleukin-17 (IL-23/IL-17) axis, which is driven by both adaptive T helper 17 (Th17) cells and group 3 innate lymphoid cells (ILC3s). These cell populations share the ability to produce IL-17A, IL-17F, and IL-22, cytokines that activate keratinocytes, promote epidermal hyperproliferation, and sustain the inflammatory microenvironment characteristic of psoriatic lesions. While Th17 cells arise from antigen-driven adaptive immune responses and contribute to the persistence of chronic inflammation, ILC3s respond rapidly to cytokine signals such as IL-23 and provide an early, antigen-independent source of IL-17 and IL-22. The functional overlap and extensive crosstalk between these two cell types create a robust and self-amplifying inflammatory circuit. Increasing evidence suggests that psoriasis comprises immunological endotypes in which either Th17 cells or ILC3s may predominate, potentially contributing to clinical heterogeneity and differential responses to targeted therapies. Because psoriasis exemplifies the cooperation between innate and adaptive IL-17-producing lymphocytes, it provides a valuable model for understanding immune regulation in chronic inflammatory and autoimmune diseases. Elucidating the balance, redundancy, and plasticity between Th17 cells and ILC3s may therefore help refine disease stratification and guide future precision-based therapeutic strategies.

## Introduction

**Figure 1:**
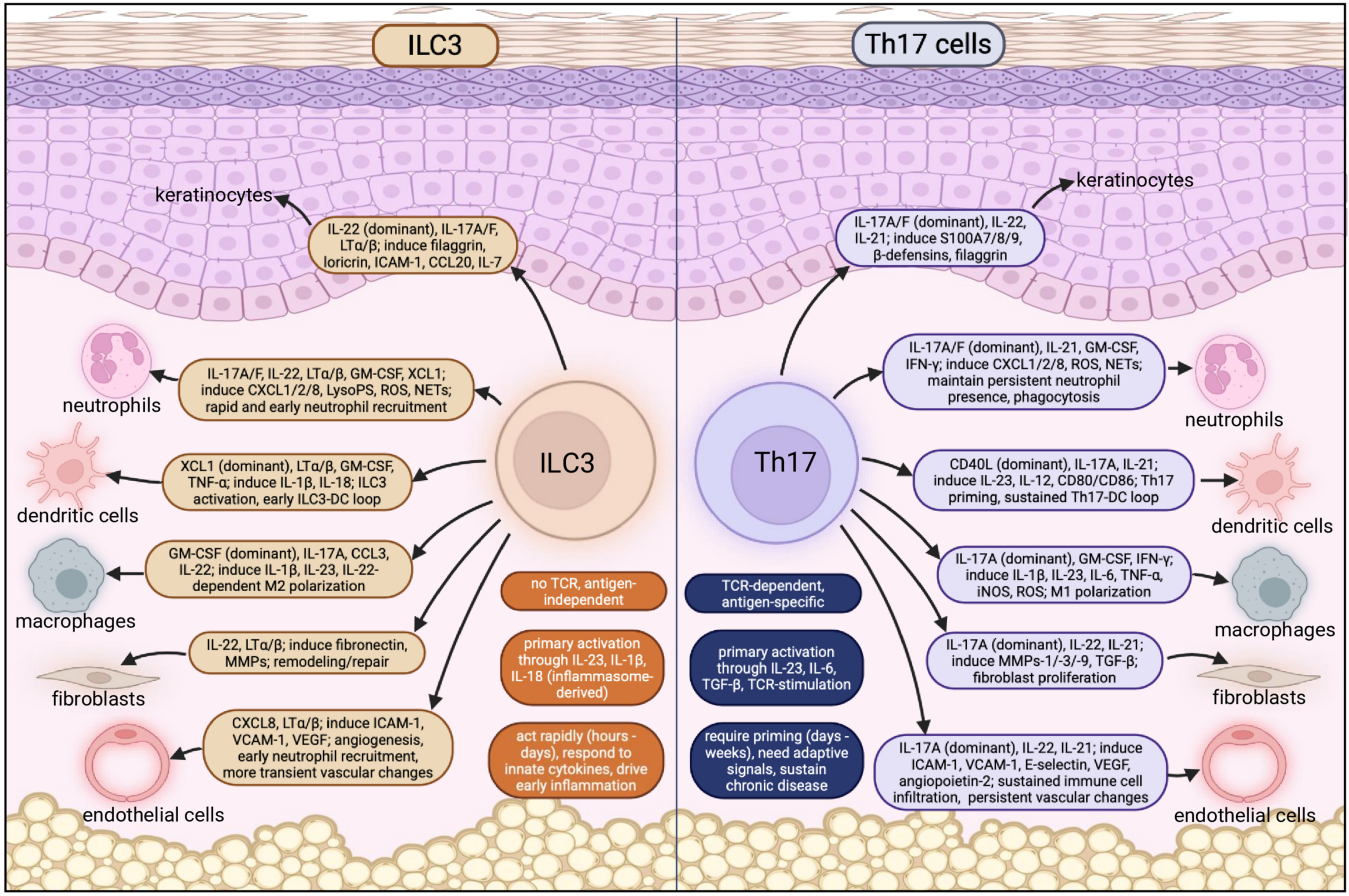
Selected interactions of ILC3s and Th17 cells with other skin cell types in the skin with relevance to the pathophysiology of psoriasis. While there are significant functional overlaps between the two cell types, there are also some distinct characteristics that at least partially differentiate the activities of ILC3 and Th17 cells and form the basis for different endotypes in psoriasis. Epidermal keratinocytes recruit ILC3s *via* CCL20 and sustain them with IL-7. ILC3-derived IL-22/IL-17 drive psoriatic plaque formation *via* keratinocyte hyperproliferation. Once activated, ILC3s produce TNF-α, lymphotoxins (LT), and lymphotactin (XCL1), a small cytokine which recruits and stimulates dendritic cells (DCs) to release IL-18 and IL-23, further amplifying ILC3 cytokine secretion. Additionally, apoptotic neutrophils release lysophosphatidylserine (LysoPS), activating GPR34-expressing ILC3s. In skin conditions like psoriasis, these activated ILC3s then secrete IL-17, IL-22, and CCL3, promoting inflammatory responses and recruitment of other cells of the immune system. There also is a macrophage-ILC3 loop, in which macrophages activate ILC3s *via* IL-1β, while ILC3s sustain macrophage inflammation through GM-CSF and IL-17. In resolution phases, ILC3-derived IL-22 may promote M2 macrophage polarization (tissue repair). ILC3s also promote fibroblast proliferation and matrix remodeling (*via* MMPs, TGF-β), contributing to chronic inflammation. ILC3s enhance leukocyte trafficking (*via* ICAM-1 and VCAM-1 on endothelial cells) and angiogenesis (*via* LTα/β, VEGF), facilitating immune cell infiltration. Th17 cells, on the other hand, drive psoriatic inflammation primarily through IL-17A, IL-17F, IL-22, and GM-CSF. The net effect is keratinocyte activation, hyperproliferation, and barrier dysfunction in the epidermis. In the dermis, they facilitate tissue remodeling and angiogenesis through activated fibroblasts and endothelial cells. Similar to ILC3s, Th17 cells create feedback loops with DCs/macrophages, facilitating chronic immune activation.

T helper 17 (Th17) cells and group 3 innate lymphoid cells (ILC3s) both contribute to IL-17-driven inflammation in psoriasis, with ILC3s providing a rapid, innate source of IL-17 and Th17 cells sustaining chronic inflammation through adaptive immune mechanisms (1–4) (**Figure 1**). In fact, it appears that the interplay between these two types of immune cells is central to the pathophysiology of psoriasis. In recent years, both cell types have been very well characterized based on numerous phenotypic features and expression patterns, and in some cases, subgroups have also been identified (**Table 1**). Both cell types are major producers of interleukin-17 (IL-17), a cytokine that drives keratinocyte activation, epidermal hyperproliferation, and the recruitment of additional inflammatory cells, establishing a self-amplifying inflammatory loop in psoriatic lesions (1, 6, 7). Th17 cells are adaptive immune cells whose differentiation and maintenance are driven by IL-23 produced by dendritic cells and macrophages. Th17 cells secrete IL-17A, IL-17F, and IL-22, which act on keratinocytes to perpetuate inflammation and tissue remodeling (1, 2, 8). ILC3s are innate immune cells that also respond to IL-23 and are capable of rapid IL-17 and IL-22 production independent of antigen stimulation. In psoriatic skin, ILC3s are activated by IL-23 and other local signals, contributing to the early and sustained production of IL-17, particularly in the initial phases of lesion development or in response to environmental triggers (3, 9).

**Table 1:** Phenotypes and developmental pathways of Th17 cells and ILC3.

Criterion	Th17 cells	ILC3
Origin	Adaptive immunity (CD4^+^ T cells)	Innate immunity (from CLP: common lymphoid progenitor)
Key transcription factors	RORγt, STAT3, BATF, IRF4	RORγt, T-bet (in ILC1/3 transitions), AHR, Id2
Surface markers	CD4^+^, CCR6^+^, CCR4^+^, IL-23R^+^, CD161^+^	Lin^-^ (CD3^-^, CD19^-^, CD14^-^), CD127^+^, CD161^+^, NCR^+^ (NKp44), CCR6^+^, CCR7^-^
Subtypes	Pathogenic Th17 (IL-17^+^IFN-γ^+^), non-pathogenic Th17 (IL-17^+^IL-10^+^)	NCR^+^ ILC3 (IL-22^+^), NCR^-^ ILC3 (IL-17^+^), LTi-like ILC3 (MHCII^+^)
Differentiation	Antigen-dependent (TCR stimulation + IL-6, TGF-β, IL-23, IL-1β)	Antigen-independent (IL-23, IL-1β, IL-7, TGF-β)
Plasticity	Conversion to Th1/Th22 upon IL-12/IL-23 withdrawal	Conversion from ILC2 → ILC3 (IL-1β, IL-23, TGF-β) or ILC1 → ILC3

Summary overview comparing the origin, markers, transcription factors, and differentiation pathways of both cell types (2, 3, 5).

Indeed, there is both significant crosstalk and redundancy between Th17 cells and ILC3s. Both cell types are recruited and activated in psoriatic plaques, and their cytokine products (especially IL-17A and IL-22) have overlapping effects on keratinocytes and the inflammatory milieu. This redundancy ensures robust IL-17 production even if one pathway is inhibited, which may explain the efficacy of IL-17 and IL-23 blockade in psoriasis therapy (1, 2, 6, 7).

## Functions of ILC3 vs. Th17 cells determine immunological tipping points

Several immunological tipping points seem to regulate the balance between Th17 cells and ILC3s, both in general inflammatory contexts and specifically in psoriasis. These tipping points involve temporal dynamics, cytokine milieu, antigen presentation, and competition for resources.

Arguably, the most fundamental temporal tipping point in psoriasis is the transition from early innate to chronic adaptive immunity. ILC3s respond rapidly to environmental stress signals without requiring antigen-specific receptors, orchestrating early immune responses within hours to days (5). As inflammation becomes chronic, antigen-specific Th17 cells are recruited and expanded, eventually becoming the dominant IL-17 source. In psoriasis, this is reflected in the observation that tissue-resident ILCs are necessary and sufficient to drive early pathology in mouse models, but during treatment-induced remission, resolved plaques contain tissue-resident memory T cells (CD8^+^IL-17^+^ or CD8^+^IFN-γ^+^ cells) that can reactivate disease (1, 3, 10).

Another critical tipping point involves ILC plasticity or ILC state transitions, respectively. Single-cell RNA-sequencing studies demonstrate that skin ILCs exist on a transcriptional continuum even at steady state. Upon IL-23 or imiquimod exposure in experimental models (11, 12), this continuum rapidly shifts toward a pathogenic ILC3-like state through multiple trajectories - including conversion from quiescent-like states and ILC2 effector states (3). This plasticity means that the inflammatory cytokine milieu (particularly IL-23, IL-1β) can rapidly reconfigure the ILC landscape toward IL-17 production.

ILC3s expressing MHC class II represent a key regulatory tipping point with context-dependent outcomes, actually constituting a bidirectional regulatory switch: Regarding tolerogenic functions in the gut, MHCII^+^ ILC3s present microbial antigens to promote RORγt^+^ regulatory T cells (Tregs) while preventing expansion of inflammatory Th17 cells. This occurs through antigen presentation, αV integrin signaling, and competition for IL-2. When this ILC3-Treg interaction is impaired, the balance may tip toward Th17 expansion and inflammatory bowel disease (13). Under inflammatory conditions (IL-1β, IFN-γ), ILC3s upregulate costimulatory molecules and can activate CD4^+^ T cells, promoting rather than suppressing adaptive responses. IL-23 suppresses MHC class II expression on intestinal ILC3s *via* mTORC1 and STAT3, reducing their tolerogenic capacity (14–16). Although there is no direct evidence of this (yet), it is conceivable that similar mechanisms also play a role in psoriasis.

## Competition for IL-2 between ILC3s and Th17 cells

ILC3s and T cells compete for IL-2, thus creating a resource-based tipping point. ILC3s can consume IL-2 that would otherwise support Th17 expansion, thereby favoring Treg differentiation over inflammatory T cell responses (13). The source of IL-2 also seems to matter - dendritic cell-derived IL-2 amplifies Tregs, while B cell-derived IL-2 induces different cellular circuits (17).

In psoriasis specifically, three interlinked inflammatory circuits create tipping points between disease phenotypes: The IL-17/IL-23/CCL20 circuit (Th17/ILC3-driven) is dominant in plaque psoriasis, while the IL-36/neutrophil axis is dominant in pustular psoriasis (18, 19). Moreover, a type I/II interferon circuit (IFN-γ, plasmacytoid DCs) is thought to play a central role in paradoxical psoriasis (20).

These circuits amplify each other through feed-forward mechanisms: IFN-γ facilitates IL-23 and Th17 responses, while IL-17 promotes IL-36 expression. The balance between these circuits helps explain clinical heterogeneity and may determine whether ILC3s or Th17 cells predominate in the pathophysiology of psoriasis (1).

## Multiple layers of complexity in the regulation of IL-17

The control of IL-17 production - notwithstanding the correlations described above - is not straightforward, but rather subject to complex regulatory mechanisms. While these cannot be fully described here, I will at least discuss some mechanisms involving Th17 cells and ILC3. In particular, IL-23-independent production of IL-17 is well-established and occurs primarily through innate and innate-like lymphocytes, including ILC3s, gamma delta (γδ) T cells, mucosal-associated invariant T (MAIT) cells, and natural killer T (NKT) cells. This pathway is regulated by alternative cytokine signals particularly IL-1β, IL-18, and IL-12 - rather than the classical IL-23-dependent mechanism that drives Th17 cell IL-17 production (21–24). The IL-23-independent pathway is primarily driven by inflammasome-derived cytokines. IL-1β and IL-18, processed by caspase-1 in the inflammasome, synergize with IL-12 or with T cell receptor (TCR) signals to induce IL-17A and IL-17F production from innate-like lymphocytes (22, 24, 25). In MAIT cells, ILC3s, and γδ T cells, optimal IL-17 production requires TCR triggering combined with IL-18 and monocyte-derived IL-12, independently of IL-23 signaling (22). IL-18 also drives ILC3 proliferation and promotes IL-22 production *via* NF-κB activation (26). Notably, some innate αβ T cells can produce IL-17 in response to TCR and IL-1 receptor ligation independently of STAT3 signaling, representing a noncanonical pathway (27). While Th17 cells classically require IL-23 for maintenance and pathogenic function, they can also produce IL-17 in a TCR-independent manner when stimulated by IL-1β or IL-18 in combination with IL-23 or IL-12 (23). ILC3s, which express RORγt and lack antigen receptors, are capable of IL-23-independent IL-17A and IL-17F production, particularly when stimulated by IL-1β and IL-18 (22, 28). Additionally, c-Kit+ ILC2s can convert into IL-17-producing ILC3-like cells in response to IL-1β and IL-23, with TGF-β promoting this conversion by inducing IL-23R and RORγt expression (29).

In psoriasis, the IL-23/IL-17 axis is central to the pathogenesis, but IL-23-independent IL-17 production contributes to disease heterogeneity and may explain differential responses to therapy (1, 2, 30). Both IL-17A and IL-17F are overexpressed in psoriatic lesions, and dual inhibition of IL-17A and IL-17F results in greater suppression of inflammatory proteins than IL-17A blockade alone (21, 22). The existence of IL-23-independent IL-17 sources - particularly ILC3s and γδ T cells - may explain why some patients respond better to IL-17 inhibitors than to IL-23 inhibitors (2, 30). The therapeutic disconnect between IL-17 and IL-23 inhibition is most striking in axial spondyloarthritis (axSpA), where IL-17 inhibitors are effective but IL-23 inhibitors have failed in clinical trials (31–33). This is attributed to the predominance of IL-23-independent IL-17 production by innate and innate-like lymphocytes at entheseal and axial sites (32, 34, 35). In contrast, psoriasis - where Th17 cells are more prominent - responds well to both IL-17 and IL-23 inhibitors, though dual IL-17A/F inhibition may provide superior outcomes in patients with significant ILC3-driven disease (21, 30).

## Antigen presenting molecules and their roles on ILC3 and Th17 cell functions in psoriasis

CD1 molecules play a significant role in the pathophysiology of psoriasis by presenting lipid antigens to T cells, thus driving IL-17 and IL-22 production. This mechanism is primarily relevant for CD1-restricted T cells (including Th17-like cells), while ILC3s have a distinct relationship with CD1 - they can express CD1d and present lipid antigens to other cells, but their IL-17 production is not dependent on CD1-mediated antigen recognition. Several CD1 isoforms arguably contribute to psoriasis pathogenesis: Langerhans cell-expressed CD1a presents lipid antigens to CD1a-restricted T cells. The latter then produce IL-17A and IL-22, contributing to psoriatic inflammation. In both mouse models and human psoriasis, CD1a amplifies Th17-mediated inflammatory responses, and blocking antibodies to CD1a alleviate skin inflammation (36–38). CD1b-autoreactive T cells are increased in patients with psoriasis, and in mouse models; these cells drive a Th17-biased cytokine response with neutrophil infiltration that is ameliorated by anti-IL-17A treatment (39). CD1d, finally, is overexpressed by keratinocytes in psoriatic plaques and can activate NKT cells to produce IFN-γ, creating a positive feedback loop that may contribute to disease pathogenesis (40).

Several specific lipid antigens presented by CD1 molecules may contribute to the Th17-related pathogenesis of psoriasis. In psoriatic lesions, mast cells release exosomes containing cytoplasmic phospholipase A2 (PLA2G4D), which transfers phospholipase activity to CD1a-expressing Langerhans cells. This generates neolipid antigens (lysophospholipids and free fatty acids) that are recognized by CD1a-reactive T cells, leading to IL-17A and IL-22 production (36, 41). Lysophosphatidylcholine (LPC), a self-lipid antigen recognized by CD1a-autoreactive T cells, has been linked to Group A *Streptococcus* (GAS)-induced psoriasis. GAS infection promotes the proliferation and activation of CD1a-autoreactive T cells that recognize LPC, providing a mechanistic link between streptococcal infection and psoriasis (42). Moreover, in hyperlipidemic conditions, phospholipids and cholesterol accumulate in diseased skin and directly activate CD1b-autoreactive T cells, potentially linking dyslipidemia to psoriasis (39).

Through another mechanism, CD1 molecules may also play a role in the function of ILC3s in psoriasis, that differs from conventional T cells. Murine ILC3s from various tissues express CD1d, with NCR (Natural Cytotoxicity Receptors)^-^/CCR (CC Chemokine Receptor)6^+^ ILC3s showing the highest levels. ILC3s can acquire lipids and load them onto CD1d to present antigens to invariant NKT (iNKT) cells (43). Although this is conceivable, there is currently no direct evidence that ILC3s presenting antigens *via* CD1d specifically induce IL-17 production by Th17 cells in psoriasis. Conversely, engagement of CD1d on ILC3s - either *in vitro* or through lipid antigen administration *in vivo* - induces ILC3 activation and IL-22 production (not IL-17). This represents a bidirectional interaction where ILC3s both present lipid antigens and respond to CD1d engagement (43). The IL-17 production by ILC3s is likely independent of CD1. Unlike CD1-restricted T cells, ILC3s produce IL-17 through cytokine-driven pathways (IL-1β, IL-23, IL-18) rather than through CD1-mediated antigen recognition. ILC3s lack antigen-specific receptors and respond to environmental stress signals and cytokines (5, 15).

The CD1-lipid antigen pathway represents a potential therapeutic target in psoriasis. PLA2 inhibition or CD1a blockade may have therapeutic potential, particularly in patients with Th17-dominant disease where CD1a-restricted T cells contribute significantly to IL-17 production (36, 37). The link between streptococcal infection, CD1a-autoreactive T cells, and psoriasis also suggests that targeting this pathway could be relevant for post-streptococcal psoriasis subtypes (42).

ILC3s, particularly the lymphoid tissue inducer (LTi)-like subset, express MHC class II and can directly present peptide antigens to CD4^+^ T cells (14, 15). This function is regulated by the transcriptional activator CIITA through a pathway similar to thymic epithelial cells (15). Upon IL-1β stimulation, peripheral ILC3s upregulate MHC class II and costimulatory molecules, enabling them to process protein antigens and prime CD4^+^ T cell responses *in vitro* and *in vivo* (14). Importantly, this ILC3–CD4^+^ T cell interaction is bidirectional: cognate interaction leads to T cell proliferation while simultaneously activating ILC3s (14, 44).

However, the functional outcome of MHC class II-mediated antigen presentation by ILC3s is context-dependent and there are several examples where it is tolerogenic. In the gut, microbiota-induced IL-23 suppresses MHC class II expression on ILC3s *via* mTORC1 and STAT3, reducing their capacity to present antigens and thereby limiting T cell responses (16). In the airway, MHC class II^+^ ILC3s restrict rather than promote Th17 cell responses. In a house dust mite model, MHC class II^+^ ILC3s limited both Th2 and Th17 cell responses, reducing airway inflammation (45). Under certain inflammatory conditions (IL-1β, IFN-γ), ILC3s can shift toward an immunogenic phenotype, upregulating costimulatory molecules and enhancing their capacity to activate CD4^+^ T cells (15, 46). The potential influence of MHC-mediated antigen presentation by ILC3 in psoriasis remains speculative at this point but certainly warrants further investigation.

Regarding the implications for psoriasis, the relationship between ILC3s and Th17 cells appears to be primarily parallel rather than hierarchical through antigen presentation. Both ILC3s and Th17 cells are major sources of IL-17 in psoriatic lesions, responding to IL-23 and IL-1β from the inflammatory milieu (1). ILC3s in psoriatic skin are enriched for NCR^+^ ILC3s that produce IL-22 and IL-17A in response to cytokine stimulation, not through cognate antigen presentation (9, 47) (see **Table 1** for subtypes). The plasticity of skin ILCs - including conversion of ILC2s to ILC3-like cells producing IL-17 - is driven by cytokines (IL-1β, IL-23, TGF-β) rather than antigen presentation (3, 29).

The potentially antigen-specific and also antigen-independent mechanisms through which ILC3 and TH17 cells contribute to the pathophysiology of psoriasis are thus complex overall and interconnected on multiple levels. Much research is certainly still needed to elucidate these relationships more precisely in the future. In any case, the different functions of the two cell types seem to open up the possibility that, depending on the dominance of individual signaling pathways, different endotypes of psoriasis could be distinguished. In fact, evidence has been accumulating recently that supports precisely this hypothesis. Therefore, the potential clinical significance of these endotypes will be discussed in the following.

## Endotypes of psoriasis with distinct Th17 vs. ILC3 profiles?

Growing clinical and experimental evidence suggests that Th17 cells and ILC3 may play relatively preferred, though not exclusive, pathophysiological roles in various types of psoriasis (**Table 2**). There is emerging evidence that endotypes of psoriasis exist in which either Th17 cells or ILC3s are favored, but these endotypes are not yet fully defined or routinely used in clinical practice. This heterogeneous disease displays complex immune circuits involving both adaptive (Th17) and innate (ILC3) IL-17-producing cells. The balance between these circuits may underlie clinical heterogeneity. For example, in classic plaque psoriasis, the IL-23/Th17 axis is typically dominant, with Th17 cells being the major source of IL-17A and IL-17F, especially in patients with strong adaptive immune activation and genetic susceptibility involving the IL-23/Th17 pathway (1, 6, 51). In contrast, certain patients - particularly those with early or non-lesional disease (clinically normal-appearing skin in patients with psoriasis), or with a strong innate immune signature - may show a relative predominance of ILC3s as the main IL-17/IL-22 source, as supported by increased numbers of ILC3s in non-lesional and lesional psoriatic skin compared to healthy controls (52–54). Additionally, the flexibility and plasticity of ILCs allow them to rapidly adapt to local inflammatory cues, and in some patients, ILC3s may be the primary drivers of inflammation, especially in the context of acute triggers or in the early phases of lesion development (3, 55).

**Table 2:** Involvement of Th17 cells and ILC3 in various forms of psoriasis.

Type of psoriasis	Role of Th17	Role of ILC3	Dominant cytokines
Plaque psoriasis	Main source of IL-17A/F, chronic	Supportive (early lesions)	IL-17, IL-22, IL-23
Guttate psoriasis	Dominant (streptococcal-triggered)	Low	Upregulation of IL-17A/F, less IFN-γ
Pustular psoriasis (GPP)	Clonal Th17 (IL-17A) in blood/skin	ILC3 + neutrophils (IL-36-triggered)	IL-36, IL-1, relatively lesser importance of IL-17
Palmoplantar pustulosis (PPP)	Th17 in NPPP (increase of IFN-γ)	ILC3 in PPP (IL-17/neutrophils)	IL-17, IL-36A, CXCL8
Paradoxical psoriasis	minor role, if any, Type I IFN dominate following TNF blockade	minor role, if any, Type I IFN dominate following TNF blockade	IFN-α/β, lesser importance of IL-17 and IL-23
Erythrodermic psoriasis	Th17/Th22 overlap	ILC3 increased, systemic inflammation	IL-17, IL-22, IL-4, IL-13
Psoriatic arthritis	Th17 in synovial fluid	ILC3 increased in synovial fluid	IL-17, IL-22, TNF-α

This comparative table illustrates the distinct and overlapping contributions of Th17 cells and ILC3s across major psoriasis subtypes, highlighting their cytokine profiles and pathophysiological roles (20, 48–50).

Current evidence suggests that some patients with psoriasis may have a Th17-dominant or ILC3-dominant immunopathological profile, but robust biomarkers for routine endotyping are not yet established. This immunological heterogeneity may have implications for therapeutic response to IL-17 or IL-23 blockade (21, 51). This also raises the question of whether Th17- or ILC3-dominated phenotypes might be associated with specific clinical forms of psoriasis.

In generalized pustular psoriasis (GPP), the IL-36/IL-1 axis dominates rather than the IL-17/IL-23 pathway that characterizes plaque psoriasis (18). Mutations in *IL36RN* (encoding the IL-36 receptor antagonist), *CARD14*, *AP1S3*, and *SERPINA3* provide genetic evidence for an autoinflammatory etiology (1). GPP lesions show higher IL-1 and IL-36 but lower IL-17A and IFN-γ expression compared to plaque psoriasis (18, 19). However, unopposed IL-36 signaling still promotes antigen-driven Th17 responses. Clonally expanded CD4^+^ T cells in GPP blood and skin are major IL-17A producers, suggesting that even in this IL-36-dominant form, adaptive Th17 cells contribute to pathology (48).

Palmoplantar pustulosis (PPP) shares IL-36 pathway activation with GPP, but shows distinct features. Transcriptional analysis reveals stronger IL-17 pathway and neutrophil-related gene signatures (including IL36A) in PPP compared to non-pustular palmoplantar psoriasis (NPPP), which instead shows enhanced IFN-γ/Th1 pathway activation (50).

Guttate psoriasis represents a Th17-dominant, antigen-driven phenotype. When skin-homing cutaneous lymphocyte antigen-positive (CLA^+^) T cells are co-cultured with epidermal cells and *Streptococcus pyogenes* extract, a dominant Th17 response emerges, with higher IL-17A and IL-17F than IFN-γ production (56). This response is enhanced in patients with HLA-Cw6 and those with streptococcal tonsillitis-associated flares (56). The acute, infection-triggered nature of guttate psoriasis suggests that adaptive Th17 cells predominate over ILC3s, as antigen-specific T cell responses are central to pathogenesis.

Erythrodermic psoriasis presents a unique challenge. Immunohistochemical studies show that erythrodermic psoriasis and erythrodermic atopic dermatitis demonstrate striking immunologic overlap - no significant differences in Th1, Th2, Th17, or Th22 cell populations distinguish these conditions during acute erythrodermic exacerbations (57). This convergence of immune phenotypes during severe, widespread inflammation suggests that the usual distinctions between Th17/ILC3 endotypes may become less relevant in erythrodermic states.

Paradoxical psoriasis is a distinct clinical entity that occurs in patients treated with tumor necrosis factor (TNF) inhibitors (rarely with other drugs), and its immunopathogenesis differs from classical psoriasis. In patients with Th17 cell-dominant endotypes, classical psoriasis is driven by adaptive immunity, with high Th17 activity and IL-17/IL-23 signaling. However, paradoxical psoriasis induced by TNF inhibitors is characterized by a shift toward innate immune activation, particularly a type I interferon signature, with prominent plasmacytoid dendritic cell (pDC) infiltration and reduced T-cell involvement, regardless of the underlying Th17 or ILC3 dominance prior to therapy (20, 58–60).

Patients with ILC3-dominant endotypes may be predisposed to paradoxical psoriasis due to their innate immune bias, but paradoxical psoriasis itself is not driven by ILC3s or Th17 cells. Instead, TNF blockade disrupts pDC maturation, leading to persistent type I interferon production and a psoriasiform eruption that is clinically similar to classical psoriasis but immunologically distinct—lacking T-cell memory and relapses (20, 58, 59). Genetic factors, such as SNPs in *IL23R*, *TNF*, and other immune-related genes, may increase susceptibility to paradoxical psoriasis, but these do not specifically distinguish Th17- from ILC3-dominant patients (61, 62). The clinical presentation appears to be similar across endotypes, with new-onset or worsening psoriasiform lesions, often within the first year of TNF inhibitor therapy (60 63, 64).

## Therapeutic implications of Th17- *versus* ILC3-dominant endotypes

Psoriasis patients exhibit molecular and cellular heterogeneity, with some endotypes dominated by Th17 cells and others by ILC3s, which may influence therapeutic response (30, 55, 76). Th17-dominant endotypes generally respond well to IL-23 and IL-17 inhibitors, with these therapies demonstrating higher rates of skin clearance and normalization of inflammatory markers compared to anti-TNF agents (1, 8, 65–68). In contrast, ILC3-dominant or stroma-proliferation endotypes may respond better to IL-17 receptor inhibitors (e.g., brodalumab) and TNF-α inhibitors, while showing less improvement with methotrexate or IL-12/23 inhibitors (76). These differential responses are supported by molecular stratification studies, which show that immune activation (Th17-driven) subtypes have robust responses to IL-23 and IL-17 blockade, whereas stroma-proliferation (potentially ILC3-driven) subtypes may benefit more from therapies targeting the IL-17 receptor or TNF-α (76).

Personalized treatment approaches using molecular stratification and biomarkers are emerging but are not yet standard in clinical practice; further research is needed to validate endotype-specific therapy selection and to integrate these findings into routine management (69).

## Comorbid diseases in different psoriasis endotypes

There is accumulating evidence that the ILC3- and Th17-related phenotypes of psoriasis are associated with different patterns of comorbid diseases. In part, this could be related to preferred functional interactions between these two cell types and resident cells, which have also been identified in the skin (**Table 3**). Patients with Th17 cell-dominant endotypes are more strongly associated with systemic inflammatory comorbid conditions, particularly cardiovascular disease, metabolic syndrome, obesity, diabetes, and non-alcoholic fatty liver disease. This is due to the central role of the IL-23/Th17 axis in driving both cutaneous and systemic inflammation, with elevated IL-17 and IL-23 levels found in atherosclerotic plaques and implicated in myocardial damage, stroke, and β-cell dysfunction in diabetes (70, 71). The expansion of Th17 cells in adipose tissue and increased IL-23 in obesity further support this link (70).

**Table 3:** Comparison of the cytokine profiles of ILC3 and Th17 cells and their effects on keratinocytes, endothelial cells, fibroblasts and dendritic cells.

Target cell	Th17 effect	ILC3 effect
Keratinocytes	Hyperproliferation (IL-17/IL-22)	Increase of IL-22, barrier gene expression
Endothelium	Adhesion molecules (upregulation of ICAM-1, VCAM-1)	Angiogenesis (upregulation of VEGF)
Fibroblasts	Fibrosis (upregulation of TGF-β)	Matrix remodeling (upregulation of MMPs)
Dendritic cells	Increased IL-23 production (feedback loop)	IL-23 consumption (competition for IL-2)

The table shows selected key functions that are relatively more pronounced in the respective cell types, although there is considerable overlap in some functions as detailed in the text (1, 6, 47). In brief, common cytokines primarily include IL-17A/F, which activate keratinocytes by upregulating ICAM-1, CCL20, and β-defensin-2, recruit neutrophils *via* CXCL1 and CXCL8, and contribute to synovial inflammation in psoriatic arthritis. Both cell types also produce IL-22, which stimulates keratinocyte proliferation *via* STAT3 activation, influences the barrier function by inducing filaggrin and loricrin, and exerts an anti-apoptotic effect by inducing Bcl-2. In contrast, IFN-γ (pathogenic Th17/Th1 transition) is more Th17-specific; it contributes to macrophage activation and M1 polarization and stimulates IL-23 production in a feedback loop. GM-CSF, which is also secreted by both cell types, recruits dendritic cells. IL-22, on the other hand, is secreted more strongly by ILC3, as are CXCL8 and lymphotoxins (LTα/β).

In contrast, ILC3-dominant endotypes appear to be more closely associated with joint involvement and psoriatic arthritis. ILC3s are enriched in the synovial fluid and blood of patients with psoriatic arthritis, where they produce high levels of IL-17A and IL-22, contributing to joint inflammation and bone damage (47, 49). The ratio of ILC2 to ILC3 correlates with the severity of joint disease, and ILC3s are implicated in the propagation of inflammation at distant sites, such as the joints, rather than systemic metabolic comorbidity (49).

## Patterns of inflammation in Th17- *versus* ILC3-dominant endotypes of psoriasis

While no single biomarker is currently validated to reliably distinguish Th17 cell-dominant from ILC3-dominant endotypes in patients with psoriasis, several promising approaches have emerged from recent translational and single-cell studies.

Single-cell RNA sequencing of lesional skin can identify immune cell transcriptomic profiles, distinguishing Th17 cell subsets (e.g., IL-17A++, IL-17F++, IFN-γ++) and their activation states, as well as ILC3 populations, but this technology is not yet practical for routine clinical stratification (67, 72, 73). Flow cytometry of peripheral blood can quantify pathogenic Th17 cells (e.g., CD4++RORγt++IFN-γ++), which correlates with disease severity and may inform biologic selection, but does not directly measure ILC3s (51, 74). Transcriptomic profiling of skin biopsy specimens can identify immune activation (Th17-dominant) *versus* stroma-proliferation (potentially ILC3-dominant) subtypes, with gene expression signatures (e.g., CCL20, IL-6, CXCL8) serving as candidate predictors for therapeutic response (75, 76). Tape strip profiling, microneedle patches, and molecular profiling from epidermal curettage are emerging noninvasive or minimally invasive methods for immune biomarker assessment, but their ability to distinguish Th17 from ILC3 dominance remains investigational (77).

It appears that this is an area where much more research will be needed in the future, particularly with regard to predicting treatment response and individual disease progression.

## Th17- and ILC3-related endotypes in early onset vs late onset psoriasis

Th17 cell-dominant endotypes are more prevalent and pronounced in early-onset psoriasis, while late-onset psoriasis may have a greater contribution from ILC3s and innate immune pathways, although both endotypes can be present across the age spectrum and contribute to disease heterogeneity. Early-onset (juvenile) psoriasis is characterized by a higher prevalence of Th17 cell-dominant endotypes, while late-onset psoriasis may show a relatively greater involvement of ILC3-dominated or mixed endotypes. In early-onset psoriasis, there is a strong genetic predisposition, more robust lymphocytic infiltrate, and a predominance of adaptive immune responses, particularly Th17 cells and their associated cytokines (IL-17A, IL-17F, and IL-22) (78–80). Pediatric psoriasis lesions, however, may display a unique cytokine profile with increased IL-22-producing T cells and relatively less IL-17 compared to adult lesions, suggesting age-related differences in Th17 cell activity and plasticity (81).

Late-onset psoriasis, which typically presents after age 40, is associated with a higher epidermal CD4:CD8 ratio and less pronounced lymphocytic infiltration, indicating a possible shift toward innate immune mechanisms, including ILC3s and other non-T cell sources of IL-17 and IL-22 (55, 78). Additionally, late-onset patients have a lower familial aggregation and are more likely to have comorbid diseases such as type 2 diabetes and autoimmune thyroiditis, which may reflect differences in underlying immune endotypes (78).

Considering the current clinical situation, there are still some unmet needs in both pediatric and elderly populations regarding endotype-based approaches to the management and treatment of psoriasis, specifically in relation to T helper 17 cell-dominant and group 3 innate lymphoid cell-dominant endotypes (82, 83). In pediatric psoriasis, the immunopathogenesis is often Th17 cell-dominant, but children may exhibit unique cytokine profiles and immune cell plasticity, complicating the extrapolation of adult data to this group. There is a lack of validated biomarkers and clinical tools for endotype stratification in children, and most biologic therapies targeting IL-17 and IL-23 are approved only for older children or adolescents, limiting precision medicine approaches and early intervention strategies (1, 82, 84). Safety, long-term efficacy, and optimal dosing in pediatric patients remain insufficiently studied, and there is a need for age-specific research on immune endotypes and their impact on comorbidity and treatment response (1, 6, 77). In elderly patients, psoriasis may present with more comorbid conditions and altered immune responses, potentially involving a greater contribution from ILC3s and innate pathways. However, there is a lack of data on how age-related immune changes affect endotype prevalence, therapeutic efficacy, and safety profiles. Elderly patients are underrepresented in clinical trials, and there is limited evidence to guide endotype-based therapy selection, especially in the context of polypharmacy and multimorbidity (1, 85).

## Future research needs

The major future research needs regarding the Th17 cell-dominant and ILC3-dominant endotypes in psoriasis include:

The definition of robust biomarkers and clinical tools for endotype stratification will be important in the near future. There is a need for validated, accessible biomarkers—such as transcriptomic signatures, cytokine profiles, or cellular phenotyping—that can reliably distinguish Th17-dominant from ILC3-dominant psoriasis in clinical practice, enabling precision medicine approaches (3, 86, 87).

Clarifying the mechanistic interplay and plasticity between Th17 cells and ILC3s will also likely become relevant. Research should focus on understanding how these cell populations transition, interact, and contribute to disease heterogeneity, including the role of environmental triggers and genetic susceptibility in shaping endotype dominance (1, 3, 30, 55).

In order to evaluate endotype-specific therapeutic responses and long-term outcomes, comparative studies are needed to determine whether Th17- or ILC3-dominant patients respond differently to IL-17, IL-23, or other targeted therapies, and to assess the impact on comorbid diseases such as psoriatic arthritis and cardiometabolic disease (7, 53, 86).

For investigating the role of non-classical IL-17 family members and IL-23-independent pathways, future work should address the contribution of IL-17F, IL-17C, and other cytokines, as well as innate and innate-like lymphocytes, to treatment resistance and disease persistence, especially in ILC3-dominant endotypes (21, 30).

Developing personalized treatment algorithms and monitoring strategies will require integrating molecular endotyping into clinical decision-making will require prospective trials and real-world studies to optimize therapy selection, minimize adverse effects, and improve patient outcomes (65, 86).

Taken together, current data firmly establish that psoriasis is driven by a robust IL-23/IL-17-centered inflammatory network in which both adaptive Th17 cells and innate or innate-like IL-17-producing populations, including ILC3s, contribute to keratinocyte activation and disease maintenance. It is also well supported that these pathways are not uniform across all patients, that ILC3s are increased in psoriatic skin, and that molecular and cellular heterogeneity can influence inflammatory patterns and possibly therapeutic response.

By contrast, the concept of clearly separable Th17-dominant and ILC3-dominant psoriasis endotypes remains an interpretative and still partly hypothetical framework rather than a validated clinical classification. In particular, proposed links between specific endotypes, clinical subtypes, comorbidity patterns, age of onset, and differential responses to IL-17-, IL-23-, or TNF-targeted therapies require prospective confirmation.

Translation of these conceptual endotypes into clinically useful stratification will therefore require reproducible biomarkers that are feasible beyond exploratory single-cell studies, including validated tissue or minimally invasive transcriptomic signatures, cytokine profiles, cellular phenotyping panels, and markers of IL-23-dependent versus IL-23-independent IL-17 production. Ultimately, prospective longitudinal studies and biomarker-stratified therapeutic trials will be needed to determine whether such profiles predict disease course, comorbid risk, and treatment response sufficiently well to guide individualized management.
